# CRIMoClo plasmids for modular assembly and orthogonal chromosomal integration of synthetic circuits in *Escherichia coli*

**DOI:** 10.1186/s13036-019-0218-8

**Published:** 2019-11-28

**Authors:** Stefano Vecchione, Georg Fritz

**Affiliations:** LOEWE Center for Synthetic Microbiology, Philipps-University Marburg, Hans-Meerwein Str. 6, 35032 Marburg, Germany

**Keywords:** Synthetic biology, Chromosomal integration, CRIM plasmids, MoClo, CRIMoClo

## Abstract

**Background:**

Synthetic biology heavily depends on rapid and simple techniques for DNA engineering, such as Ligase Cycling Reaction (LCR), Gibson assembly and Golden Gate assembly, all of which allow for fast, multi-fragment DNA assembly. A major enhancement of Golden Gate assembly is represented by the Modular Cloning (MoClo) system that allows for simple library propagation and combinatorial construction of genetic circuits from reusable parts. Yet, one limitation of the MoClo system is that all circuits are assembled in low- and medium copy plasmids, while a rapid route to chromosomal integration is lacking. To overcome this bottleneck, here we took advantage of the conditional-replication, integration, and modular (CRIM) plasmids, which can be integrated in single copies into the chromosome of *Escherichia coli* and related bacteria by site-specific recombination at different phage attachment (*att*) sites.

**Results:**

By combining the modularity of the MoClo system with the CRIM plasmids features we created a set of 32 novel CRIMoClo plasmids and benchmarked their suitability for synthetic biology applications. Using CRIMoClo plasmids we assembled and integrated a given genetic circuit into four selected phage attachment sites. Analyzing the behavior of these circuits we found essentially identical expression levels, indicating orthogonality of the loci. Using CRIMoClo plasmids and four different reporter systems, we illustrated a framework that allows for a fast and reliable sequential integration at the four selected *att* sites. Taking advantage of four resistance cassettes the procedure did not require recombination events between each round of integration. Finally, we assembled and genomically integrated synthetic ECF σ factor/anti-σ switches with high efficiency, showing that the growth defects observed for circuits encoded on medium-copy plasmids were alleviated.

**Conclusions:**

The CRIMoClo system enables the generation of genetic circuits from reusable, MoClo-compatible parts and their integration into 4 orthogonal *att* sites into the genome of *E. coli*. Utilizing four different resistance modules the CRIMoClo system allows for easy, fast, and reliable multiple integrations. Moreover, utilizing CRIMoClo plasmids and MoClo reusable parts, we efficiently integrated and alleviated the toxicity of plasmid-borne circuits. Finally, since CRIMoClo framework allows for high flexibility, it is possible to utilize plasmid-borne and chromosomally integrated circuits simultaneously. This increases our ability to permute multiple genetic modules and allows for an easier design of complex synthetic metabolic pathways in *E. coli*.

## Introduction

Synthetic Biology aims at applying engineering principles to biological systems [1], yet the complexity of living cells often leads to unpredictable behavior of heterologous genetic circuits. For instance, the expression of synthetic circuits from medium- or high-copy plasmids in *E. coli* can lead to toxic side effects on the chassis cell, as being the result, e.g., of competition for essential cellular resources, such as metabolites, RNA polymerase or ribosomes [2]. Thus, reducing such undesired cross-reactions with the host are among the key factors for the success of a rational, model-driven design of novel synthetic circuits. While this has led to the development of a number of orthogonal (i.e. context independent) regulators for synthetic circuit construction [3–5], high plasmid copy numbers can still generate growth defects, as e.g. in the case of the expression of membrane-anchored anti-σ factors regulating the activity of orthogonal extracytoplasmic function (ECF) σ factors [6].

**Fig. 1:**
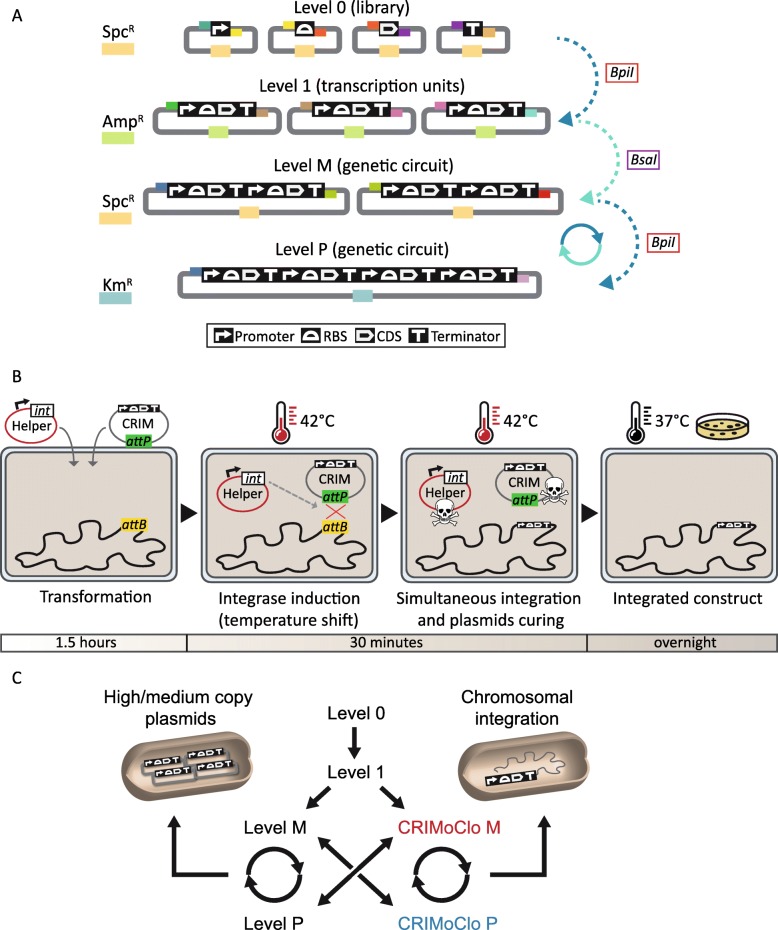
(**a**) Vector organization in the different levels of the MoClo system. Level 0 vectors are used to generate a library of parts that can be subsequently assembled in Level 1 transcription units (TU), which in turn serve to generate Level M and P multi-TU constructs. The antibiotic cassettes and the four bases overhangs (fusion sites) of each level are represented by colored boxes. The different genetic parts are represented as pictograms in black boxes. The Type IIs restriction endonucleases used for each cloning step are indicated. (**b**) Chromosomal integration framework using Conditional-replication, integration, and modular (CRIM) plasmids. The co-transformation of a CRIM-based plasmid and the cognate helper plasmid is followed by a temperature shift that induces the expression of the recombinase, driving the site-specific recombination event at the specific phage attachment site. The proprieties of the system allow for the integration and curing of the helper plasmid in the same incubation step and easy selection for recombinant clones after overnight incubation. (**c**) Joint use of CRIMoClo plasmids (Level M and P) with other vectors in the MoClo system. Any transcription unit generated in Level 1 can be cloned into high/medium copy number MoClo plasmids or be chromosomally integrated using CRIMoClo plasmids. The design of CRIMoClo plasmids allows for a seamless transition between the two systems

Besides approaches for reducing toxicity, synthetic biology relies on a comprehensive set of modular and well-characterized DNA parts as well as modular cloning strategies [7]. Currently, there are several DNA assembly methods used across the synthetic biology community [8] that are based on Gibson Assembly [9], Ligase Cycling Reaction [10], Gateway cloning [11], and Golden Gate cloning [12]. With these methodologies, DNA parts such as promoters, coding sequences, and terminators are assembled to build functional transcription units that can then be combined in more complex genetic circuits. Among them, Golden Gate cloning is based on Type IIS restriction digestion and ligation, and allows for cloning of multiple genetic parts in a one-tube reaction. A major innovation of the original Golden Gate assembly framework came with the development of the Modular Cloning (MoClo) system that enables not only hierarchical construction of multigene constructs but also full reusability of parts [13, 14]. The MoClo system provides a series of vectors that are organized within different levels (Fig. 1a). A specific antibiotic cassette and the positioning of the two different Type IIS restriction sites, that generate pre-defined 4 base pair (bp) overhangs (fusion sites), define the level of each vector. The fusions sites allow for directional cloning of multiple genetic parts, hence enabling hierarchical assembly of transcription units and genetic circuits with increasing complexity through the different levels. Simple library preparation and combinatorial assembly from reusable parts make the MoClo system a valuable tool for synthetic biology applications [15]. However, even though the system permits complex genetic circuit generation [4], to date most circuits are assembled on low- or medium-copy plasmids. When studying complex genetic circuits or biosynthetic pathways this may lead to overproduction of proteins, which can generate adverse effects on growth and stress responses that are not present at lower levels of expression [16]. Also, while low-copy plasmids may circumvent issues with protein overexpression, plasmid stability is only guaranteed under permanent antibiotic selection, which can also have negative effects on cell growth and physiology.

To circumvent these issues, different methods for integrating DNA from plasmids into the *E. coli* chromosome have been developed. For instance, recombineering-based strategies [17, 18], such as KIKO vectors [19], facilitate the integration of large circuits in *E. coli* [18]. However, these strategies often rely on traditional restriction digestion and ligation, which limit the speed of circuit construction and do not allow for recycling of genetic parts. Recently, an innovation was developed by Schindler et al., providing a series of MoClo-compatible vectors that facilitate lambda Red-based integration [20]. However, recombineering-based strategies tend to suffer from another limitation, which is the lack of well-characterized orthogonal loci. For instance, it was shown that protein expression and metabolite production in *E. coli* may be influenced by the location of their integrations sites on the genome [21] and even though five novel open reading frames were identified as suitable integration loci for synthetic circuits in *E. coli*, the integration efficiency and expression of genetic constructs in these loci varied significantly [22].

An alternative way to perform chromosomal integrations is based on bacteriophage integrases [23]. A prime example embarking on this strategy is implemented in the “conditional-replication, integration, and modular” (CRIM)-based plasmids, which carry different phage attachment (*attP*) sites and can be used to insert large DNA fragments at bacterial phage-attachment (*attB*) sites (Fig. 1b). The site-specific recombination is driven via expression of phage-derived integrase (*int*) gene encoded on a helper plasmid. The integration procedure is simple, requiring only the co-transformation of the bacterial strain with a CRIM plasmid, together the cognate helper plasmid, and a temperature shift that induces expression of the integrase. Since the helper plasmid is temperature sensitive for replication, the integration of the CRIM and the cure of the helper plasmid occur simultaneously [23]. Moreover, CRIM plasmids possess the γ conditional origin of replication of R6K that depends on the trans-acting π protein (encoded by *pir*) for replication. Hence, successful integration events can easily be selected by transforming a *pir*^−^ host with CRIM plasmids under antibiotic selection. These characteristics make CRIM plasmids a fast and reliable strategy for chromosomal integration in *E. coli,* and even though the system was further improved [24], so far CRIM-based integration methods lacked standardization, limiting the speed of DNA assembly/integration and not enabling for the reusability of genetic parts.

To fill this gap here we combine the standardization and modularity of the MoClo system, with the high integration efficiency of CRIM plasmids, generating a set of CRIMoClo plasmids (Fig. 1c). We benchmark their suitability for synthetic biology approaches assembling synthetic circuits from reusable Level 0 parts and showing the orthogonality of the four phage attachment sites and four different resistance cassettes. Further, we present a strategy that facilitates the sequential integration of different inducible reporter systems at the four phage attachment sites, showing the modularity and the efficiency of the framework. Finally, we use our fast and reliable assembly/integration strategy to perform a large multi-part assembly (∼10 kb) and integration of synthetic ECF-σ/anti-σ switches, showing that growth defects observed for circuits encoded on medium-copy plasmids are abolished.

## Results and discussion

**Fig. 2:**
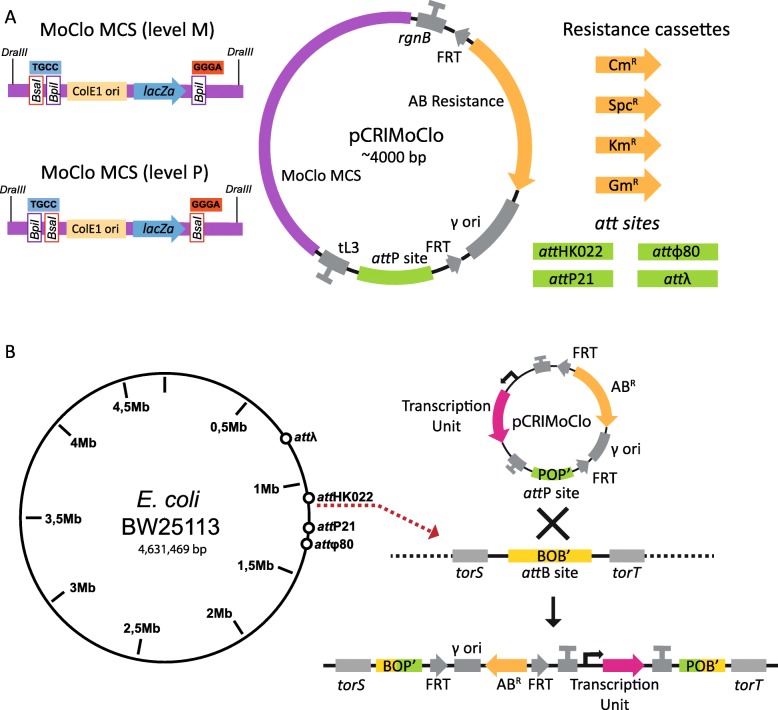
(**a**) Blueprint of CRIMoClo plasmids. Each plasmid has a MoClo-compatible cloning cassette (purple box), flanked by two terminators (*rgnB* and *tL3* depicted in grey) and followed by a selectable resistance marker (orange arrows), a γ conditional-replication origin (grey box), both enclosed between two FRT sites (grey arrows). All CRIMoClo plasmids have one of four different phage attachment sites (green boxes) for the integration in the chromosome by site-specific recombination. (**b**) Locations of chromosomal *attB* sites in the annotated genome of *E. coli* BW25113 (left) and site-specific recombination of CRIMoClo-based plasmid into the att_*HK022*_ site (right)

The design of CRIMoClo plasmids aims to combine the combinatorial features of MoClo vectors with the reliable and highly efficient chromosomal integration of CRIM plasmids. As illustrated previously, the MoClo system provides a series of vectors that are organized in different levels defined by a specific resistance cassette and different 4 nt overhangs (Fig. 1a). The properties of the MoClo system support for easy generation of a library of genetic parts that, upon PCR amplification, can be cloned in Level 0 vectors. Conforming to the characteristic of the MoClo vectors [13, 14], up to 8 Level 0 parts can be assembled in Level 1 vectors to generate functional transcription units (TUs). Up to six TUs can be combined from Level 1 to Level M, and from Level M to Level P. The system consents also a continuous assembly of parts from Level P to Level M and vice versa. Since our and other publicly available MoClo multipart libraries [15] provide genetic parts stored in Level 0 and Level 1 vectors, we generated CRIMoClo plasmids possessing Level M (pAGM8031) and Level P (pICH75322) cloning sites (Fig. 2a). This permits not only the use of CRIMoClo plasmids as integrative vectors but also as MoClo vectors, providing a seamless transition between the two systems (Fig. 1c). Within the Level M and P cloning sites we also maintained the *lacZα* fragment coding region from *E. coli* for blue/white selection and added a high copy number origin of replication (ColE1) derived from MoClo vector pICH82094 [13] (Fig. 2a). CRIMoClo plasmids also possess the γ conditional origin of replication of R6K, which requires the *trans*-acting π protein (encoded by *pir*) for replication, allowing them to replicate at a medium (15 per cell) plasmid copy number in *pir*^+^ *E. coli* hosts, but not in normal (non-*pir*) hosts [23]. The coexistence of ColE1 and R6K origins of replication did not affect the stability of the plasmids and permits propagation of the CRIMoClo vectors at high copy number before cloning a construct (relying on ColE1), and their conversion into suicide vectors after cloning (relying on R6K). This in turn enables efficient chromosomal integration of the CRIMoClo plasmids in non-*pir* hosts (see below). To insulate the insert from transcriptional read-through, in our design the MoClo cloning module is flanked by bacterial (*rgnB*) and phage λ (tL3) terminators (Fig. 2a). Last, each CRIMoClo vector exists in four variants that differ in the selectable resistance markers (Fig. 2a), supporting high flexibility while maintaining the capability of switching between Level M and Level P.

CRIM plasmids can be integrated efficiently into selected phage attachment sites on the chromosome of *E. coli* and other bacteria [23]. To maintain this feature, CRIMoClo plasmids possess one of the four phage attachment sites (*att*_HK022_, *att*_P21_, *att*_ϕ80_, *att*_λ_) that previously showed the highest integration efficiency [23] (Fig. 2a). The chromosomal integration into one of the *attB* sites follows the same strategy of CRIM plasmids (Fig. 2b), where a non-*pir E. coli* strain carrying a CRIM helper plasmid (expressing the recombinase specific to the integration into the respective chromosomal *attB* site [23]) is transformed with the CRIMoClo-based plasmid. Alternatively, CRIMoClo and helper plasmids can be efficiently co-transformed (see results below) using the transformation and storage solution (TSS) technique [25]. Independent of the preferred strategy, expression of the integrase gene encoded in the helper plasmids is induced at elevated temperatures during the transformation procedure (see Material and Methods), and since the helper plasmids are temperature-sensitive for replication, integration and curing of the helper plasmid occur in the same incubation step (Fig. 2b). Single integration events can be then screened by colony PCR with four primers (P1-P2-P3-P4; see Table 1) that enable the distinction between single, multiple, and no integration events. Like the original CRIM plasmids, CRIMoClo plasmids can also be excised efficiently from the chromosome by another round of helper plasmid transformation and *excisionase* (Xis) expression [23].

**Table 1:**
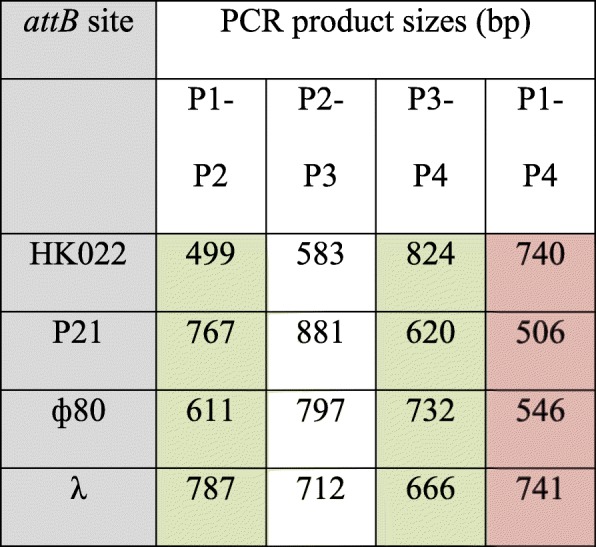
Predicted PCR product sizes for *attB* sites, using primers P1-P2-P3-P4. Successful integration events at each *attB* site are revealed by two fragments generated by P1-P2 and P3-P4 (highlighted in green). Recombinants with two (or more) CRIMoClo plasmids at the *attB* site show in addition a third fragment generated by P2-P3. False positive (non-integrants) are revealed by the PCR product generated by P1 to P4 (highlighted in red)

Based on this design we created a combinatorial set of 32 CRIMoClo plasmids featuring all permutations of four phage attachment sites (*att*_HK022_, *att*_P21_, *att*_ϕ80_, *att*_λ_), four resistance cassettes (chloramphenicol, kanamycin, spectinomycin, gentamicin) and compatibility with two MoClo Levels (M and P). In theory, this now enables efficient assembly and chromosomal integration of synthetic genetic circuits in only 4 days, starting from Level 1 transcription units (see below). Moreover, the availability of each plasmid with one of four resistance cassettes should allow, in principle, sequential integration in different *att* sites without the necessity of recombining the resistance cassette after each integration step.

### Insulation and robustness of gene expression at *attB* sites

**Fig. 3:**
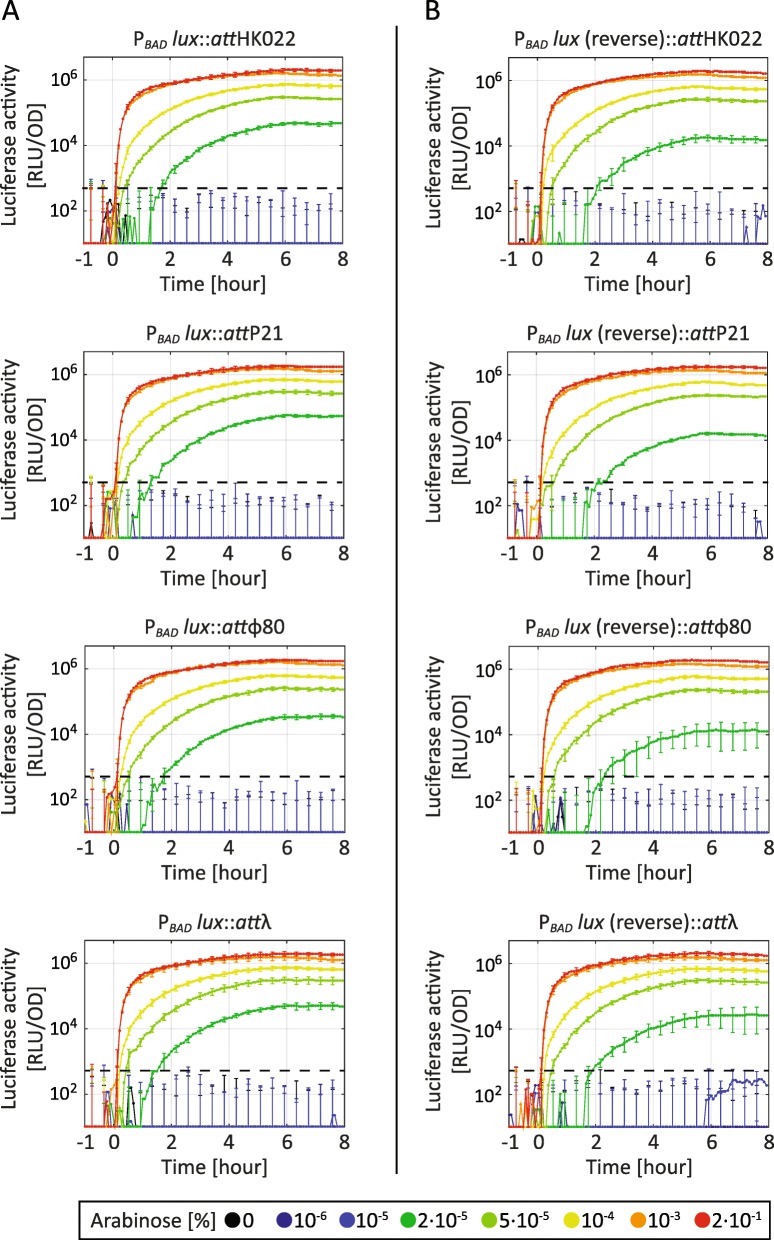
Comparison of dynamical response of luciferase activity of the P_BAD_-*lux* construct integrated into *att*_HK022_, *att*_P21_, *att*_ϕ80_, *att*_λ_ in forward (**a**) and reverse (**b**) orientation*,* after the addition of indicated concentrations of arabinose at t = 0 h. The results are averaged from at least two independent biological assays and error bars denote standard deviations. The black dashed line represents the instrument detection limit, defined as three times the standard deviation of the luminescence signal registered in a well filled with growth medium, divided by the averaged OD_600_ values registered in the same well. All signals at or below the instrument detection limit are not considered as significant

To demonstrate the versatility of CRIMoClo plasmids for synthetic biology applications, we evaluated the modularity of chromosomal integration into the four *attB* sites. To this end, we compared the expression of the same reporter constructs integrated into each of the *attB* sites. The position of the integration sites (*attB*) in the chromosome of *E. coli* BW25113 (the closest parental strain of our reporter strain (SV01) with an annotated genome) is shown in Fig. 2b. Since essential chromosomal genes lie between the different integration sites and since all CRIMoClo plasmids are integrated into the same relative orientation, recombination among them does not lead to genome instability, as previously described [23] and also demonstrated further in this work. In order to evaluate gene expression from constructs integrated into the four *attB* sites, we used a reporter construct that we previously generated in our laboratory using MoClo [4] (Additional file 1: Figure S1). This construct consists of an arabinose-inducible promoter P_*BAD*_ [26] in combination with its regulatory protein (AraC) encoded in divergent orientation, fused to the luciferase cassette from *Photorhabdus luminescens* [27], using a strong ribosome binding sequence (st8 [28]) and a strong synthetic terminator (L3S2P21) [29]. The transcription unit encoded on MoClo Level 1 vector was sub-cloned in all 16 CRIMoClo Level M plasmids (all combinations of four selected *att* sites and four antibiotic selection markers) simultaneously in 1 day. On day 2 we verified the constructs by colony PCR and started overnight cultures. On day 3 we isolated the plasmids, verified them by restriction digestion and integrated them in the four different phage attachment sites (*att*_HK022_, *att*_P21_, *att*_ϕ80_, *att*_λ_) of *E. coli* strain SV01. This strain has defined deletions of *araBAD, lacZ* and *lacI* and constitutively expresses the arabinose permease *araE* allowing a graded induction of the arabinose-inducible P_*BAD*_ promoter [4]. On day 4 we tested the clones with colony PCR (see Material and Methods) and strikingly, single integration events showed a success rate of 97% (calculated as average of positive clones screened by colony PCR) for all selected *att* sites and selection markers combined. The expression of the luciferase cassette, which has proven to be a highly sensitive reporter system for in vivo gene expression analyses [30], was benchmarked by growing strains in defined minimal media (doubling time ∼175 min) and assaying luciferase activity continuously for 8 h after the addition of the inducer (Fig. 3a). We found that in all integration loci the luciferase signal was not detectable in the absence of the inducer, while after induction, the luciferase operon was expressed from all promoters (∼10,000- to 100,000-fold over empty vector control depending on the inducer levels). Strikingly, in all integration loci, the expression dynamics (corresponding to different inducer levels) of the luciferase reporter construct was almost identical.

**Fig. 4:**
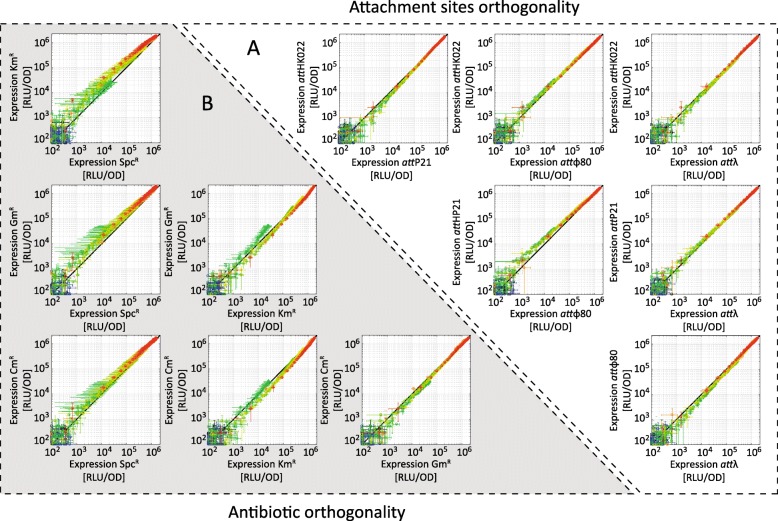
Orthogonality of reporter gene expression between different integration sites and between different resistance cassettes used for integration. (**a**) Correlation graphs between the luciferase activities in Fig. 3a, obtained from P_BAD_-*lux* integrated into different *att* sites. Each data point represents mean and standard deviation measured at the same time point in Fig. 3a, while the color code indicates the inducer concentration as in Fig. 3. (**b**) Correlation graphs between luciferase activities obtained from P_BAD_-*lux* integrated into *att*_HK022_, using CRIMoClo plasmids with four indicated resistance cassettes (chloramphenicol, kanamycin, spectinomycin, gentamicin). All data indicate averages from at least two independent biological assays and error bars denote standard deviations

The position and orientation of genes on the chromosome may affect the expression pattern of a neighboring transcription unit (TU), e.g., due to transcription-induced DNA supercoiling, affecting the activity of neighboring promoters [31]. Even though the different CRIMoClo plasmids are integrated into the same relative direction, the orientation of the cloned construct can be easily inverted using the properties of the MoClo system (e.g. starting with Level 1 parts cloned in reverse orientation). To test whether the expression of a TU is influenced by its orientation in the four selected *att* sites on the genome, we integrated P_*BAD*_-*lu*x in reverse orientation in all integration loci (Fig. 3b). The results show again no substantial difference in the expression levels of the construct from the different *att* sites. Moreover, the expression levels of constructs cloned in forward and reverse orientation are virtually identical (Fig. 3a and b), indicating that the circuits are well insulated from the genetic context and that the *att* sites are not affected by effects such DNA supercoiling. Finally, when quantitatively comparing the luciferase activities between the different integration loci, we find a striking correlation of the detected signals for each arabinose concentration at individual time points (Fig. 4a).

As a further test to compare gene expression levels from the different loci, we fused the *lux* operon to two additional inducible promoters, P_*tet*_ [32] and P_*Llac0–1*_ [33], and integrated each of these reporter constructs into the four *att* sites. The reporter constructs also contain expression cassettes encoding the relative repressors (TetR and LacI, respectively), transcribed in divergent orientation to the P_*tet*_ and P_*Llac0–1*_ promoters, respectively. For both promoter*-lux* fusions*,* quantification of reporter activity showed that the luciferase signals were independent of the integration locus, and varied in a concerted manner with the inducer level, i.e. anhydrotetracycline (ATc) for P_*tet*_ and isopropyl-β-D-thiogalactoside (IPTG) for P_*Llac0–1*_ (Additional file 1: Figure S2). Concluding, our results show that integration into the four *att* sites leads to highly reproducible gene expression behavior, suggesting that these sites can be used as interchangeable and orthogonal loci for chromosomal integration of synthetic circuits.

Since CRIMoClo plasmids exist with four different resistance cassettes (Fig. 2a), we wanted to test the potential influence of the resistance cassettes on reporter activity after chromosomal integration. To this end, we integrated and measured the expression level of P_*BAD*_-*lux* integrated into all selected *att* sites, using CRIMoClo plasmid possessing different selection markers (Fig. 4b and Additional file 1: Figure S3-S5). The results show again virtually no difference in the expression levels, suggesting that also the different resistance cassettes can be used interchangeably.

Finally, we assayed the genomic stability of the integration by using four strains harboring P_*BAD*_-*lux* constructs integrated into the four phage attachment sites (Fig. 3a). Additional file 1: Figure S6A shows that all the colonies of the four strains are luminescent when streaked onto LB plates supplied with 0.2% arabinose, in absence of antibiotic selection. Moreover, Additional file 1: Figure S6B shows that the same strains are still luminescent after being precultured for 7 days in absence of antibiotic selection.

Overall, these results show that the four phage attachment sites are well-insulated from their genomic context and that the different positions and orientations of the transcription units on the chromosome do not influence the dynamics of reporter gene expression. Moreover the four selected *att* sites guarantee a stable integration of a given genetic construct even in absence of antibiotic selection. Therefore, our experiments demonstrate orthogonality of the four phage attachment sites of the CRIMoClo system and verify the stability of integrated constructs, which makes them ideally suited for synthetic biology applications.

### Multi-locus integrations

**Fig. 5:**
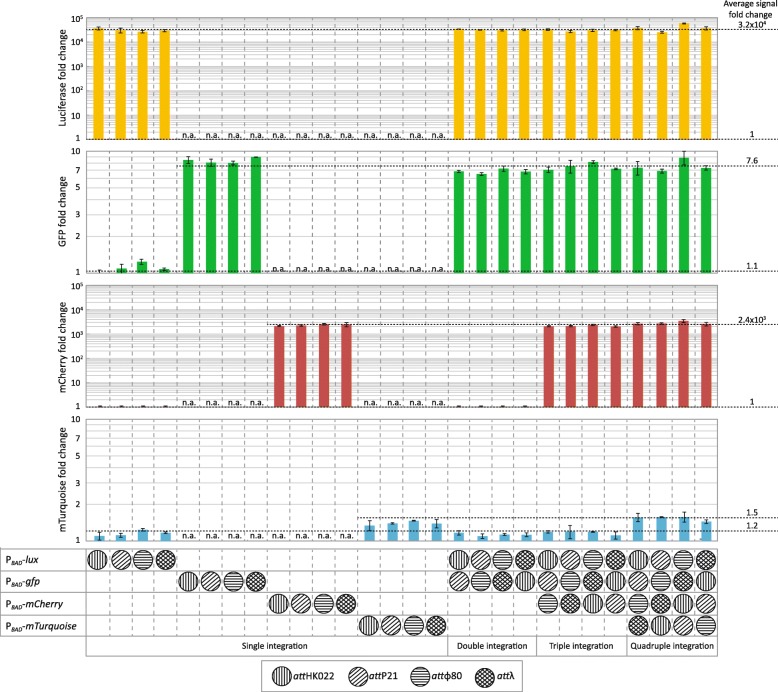
The dynamic range of four different arabinose-inducible reporter constructs, integrated sequentially into the genome of *E. coli* in four phage attachment sites (*att*_HK022_, *att*_P21_, *att*_ϕ80_, *att*_λ_) and control strains in which the same reporter systems are integrated singularly in one of four phage attachment site. The fold-change for each reporter (luciferase, GFP, mCherry, mTurquoise) is measured as the reporter signal in the presence of 0.2% of arabinose, divided by the basal activity of the reporter in the absence of arabinose. All data indicate averages from at least two independent biological assays and error bars denote standard deviations. A Student’s t-test (two-tailed, two-sample unequal variance) shows that the mTurquoise signals of the strains carrying the mTurquoise reporter are incompatible with the signals of the strains not carrying the mTurquoise reporter (*p*-value = 0.003), indicating that the signals are statistically different. The same holds for all other reporters, for which the difference between strains +/− reporter are more evident

The high efficiency of site-specific recombination combined with the availability of four different resistance cassettes should, in principle, enable fast and reliable multi-locus integrations. To demonstrate this we sequentially integrated four different reporter cassettes into the *E. coli* chromosome*,* without removing the selectable marker after each integration step. The novel transcription units were built using previously generated genetic parts (inducible P_*BAD*_ promoter, st8 ribosome binding sequence, and L3S2P21 terminator) fused with different reporter genes (*gfp*, *mCherry, mTurquoise*). To achieve sequential multi-integration, we started off with the *E. coli* strains harboring a P_*BAD*_*-lux* transcription unit inserted in *att*_HK022,_ *att*_P21,_ *att*_ϕ80,_ and *att*_λ_ that we generated and analyzed previously (Fig. 3). Subsequently, for each strain we integrated P_*BAD*_-*gfp* in the next available locus, following the above-mentioned order (*att*_HK022,_ *att*_P21,_ *att*_ϕ80,_ and *att*_λ_), as described in Methods and shown in the lower panel of Fig. 5. The newly generated double integration strains were then used for the integration of P_*BAD*_-*mCherry* in the next free *att* site (triple integrations) and subsequently of P_*BAD*_-*mTurquoise* in the last available *att* site (quadruple integrations). As a positive control for the expression levels of the fluorescence reporters in the newly generated strains, we also assembled and integrated each reporter construct (P_*BAD*_-*gfp,* P_*BAD*_-*mCherry,* P_*BAD*_-*mTurquoise*) into each of the four *att* sites alone. All the strains were monitored within time course experiments for OD and the four reporter activities (in case of multi-integration, also in the intermediate strains containing only one, two, or three reporter constructs) in presence and absence of the inducer (Additional file 1: Figures S7-S11). The histogram in Fig. 5 shows the dynamic range (reporter signal of induced strain, divided by reporter signal of uninduced strain), measured after 10 h, of all single integrant strains in comparison with strains generated with single, double, triple or quadruple integration events. Overall, we found that all the transcription units were functional after the integration (Fig. 5). The luciferase construct showed the highest dynamic range (∼3.2·10^4^-fold induction), consistent with the fact that the reporter is based on an enzymatic reaction with virtually no background activity. This dynamic range is then followed by mCherry (∼2.4·10^3^-fold induction), GFP (∼7.6-fold induction) and mTurquoise (∼1.5-fold induction). Here, the lower dynamic range observed for GFP compared to the mCherry reporter is likely due to green autofluorescence of *E. coli* cells caused by the excretion of flavins [34], thereby reducing the signal-to-noise ratio for this reporter. Likewise, mTurquoise has been reported to feature a long maturation time of 112 min [35], which can also reduce the apparent output dynamic range of this reporter. Independent of these reporter-specific dynamic ranges, we found little cross-talk among the reporters, as indicated by the fact that single, double, and triple integration strains showed only significant activity in the reporters that were present in the respective strains. Strikingly, the dynamic range values for single integrant strains are identical (within the error tolerance) to the ones calculated for single, double, triple, and quadruple integration strains (Fig. 5), showing that the sequential integration of each reporter construct does not affect the expression level of the previously integrated ones. Also, the simultaneous presence of multiple copies of P_*BAD*_ in the same strain does not lead to instability or change in the reporter expression levels, suggesting that the potential recombination among the loci does not occur. Overall, these results show that the multi-integration strategy using CRIMoClo plasmids is highly flexible and does not require time-consuming removal of resistance cassettes after the individual integration steps.

### Application of CRIMoClo plasmids to minimize heterologous expression burden

Genetic circuits encoded on plasmids can generate undesired effects on cellular physiology [36, 37]. For instance, plasmid maintenance, as well as high expression of heterologous genetic constructs, can cause a metabolic burden to the cell, and therefore toxicity [38–40]. Hence, lowering the copy number of the construct using low copy number plasmids or via chromosomal integration is often a solution. Yet, the size of the genetic circuits can often limit the efficiency of chromosomal integration and even though there are strategies that facilitate the integration of large genetic circuits [18, 24], they generally do not support easy, modular circuit generation.

**Fig. 6:**
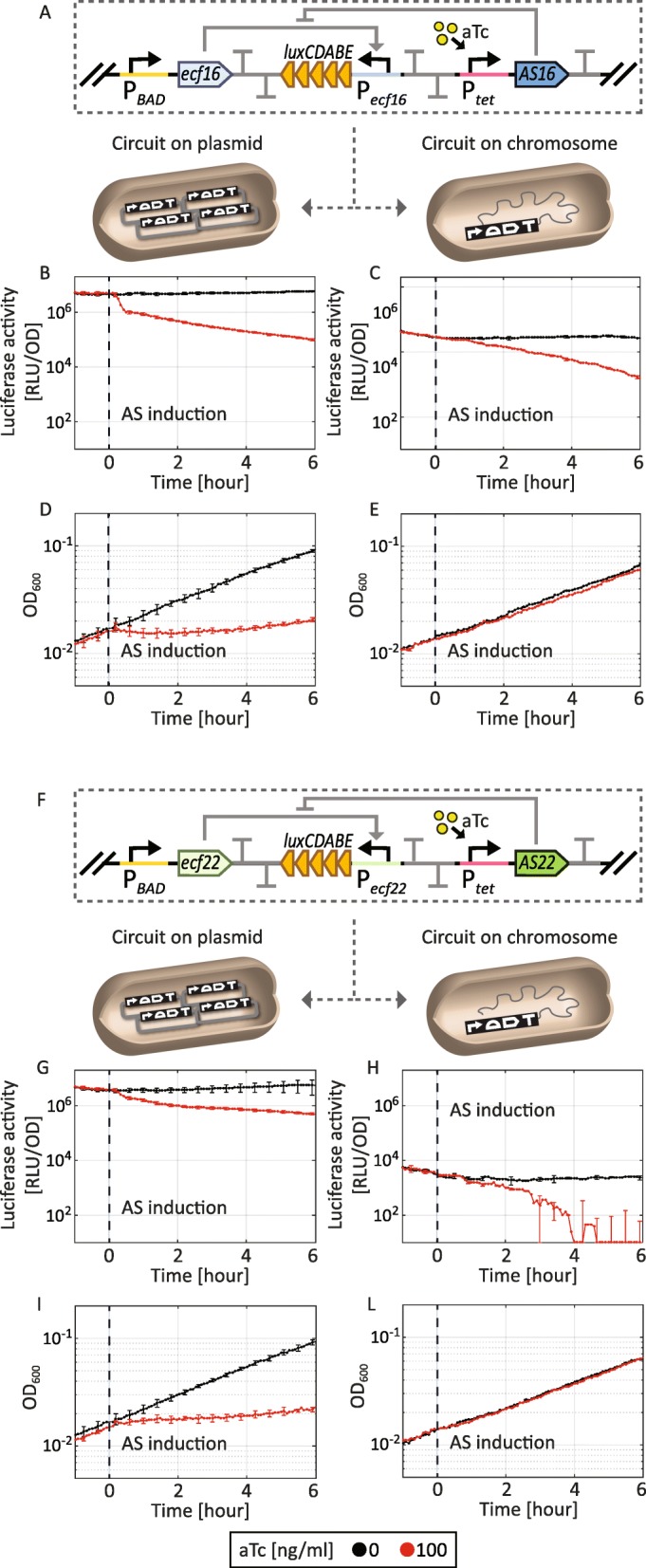
Comparison of two ECF σ/anti-σ switches encoded on medium copy plasmid and chromosomally integrated. Optical density (measured at 600 nm) and luciferase activity (shown in relative luminescence units normalized by the optical density measured at 600 nm) from an ECF16 σ-dependent promoter (**a**) and an ECF22 σ-dependent promoter (**b**), in presence or absence of the cognate anti-σ. The growth defects observed in both circuits encoded on medium copy number plasmids are abolished when integrated into the genome, while the ability of the anti-σ to sequester the cognate ECF σ is maintained. The data indicate averages from three independent biological assays and error bars denote standard deviations

Previously we used a MoClo-based strategy to build synthetic timer circuits based on alternative, extracytoplasmic function (ECF) σ factors [4]. ECFs are the smallest and simplest alternative σ factors and they have a great potential as orthogonal regulators for the design of novel synthetic circuits in *E. coli* and *B. subtilis* [4]. In order to improve the dynamic response of our ECF-based circuits here we wanted to introduce anti-σ (AS) factors that inactivate ECFs by sequestration and thereby can lead to a more clear-cut switching from the off- to the on-state of an ECF switch. However, AS factors are often transmembrane proteins that can become toxic when expressed at a high level in *E. coli* [6]. Being aware of this possible issue and in order to use AS factors in our genetic circuits, we wanted to benchmark their toxicity as well as their ability to sequester the cognate ECF σ factor. To do so, we wanted to compare toxicity and functionality of the same AS-based circuits encoded on medium copy number plasmids or chromosomally integrated. Hence, we used the medium copy number MoClo compatible vector pSVM-mc [4] and CRIMoClo plasmids as destination vectors to assemble 11 ECF-σ/anti-σ switches (Fig. 6a,f). All switches include two inducible promoters, P_*BAD*_ and P_*tet*_, controlling the expression of an ECF σ factor and its cognate AS, respectively. Moreover, the relative ECF target promoter is fused to a luciferase cassette, used to assay ECF σ factor activity. Using the MoClo we successfully assembled 22 circuits in parallel (11 ECF σ/anti-σ switches on medium copy number MoClo vector and CRIMoClo *att*_HK022_) and even though the size of these circuits is ∼10 kb, overall, we observed a high chromosomal integration efficiency (all clones were positive when one per construct was tested via colony PCR and restriction digestion). Figure 6 shows the functionality and toxicity effect of two ECF σ/anti-σ switches in detail. In our previous analysis [4] we observed that for these particular ECFs, the basal activity of the P_*BAD*_ promoter is sufficient to produce a number of ECFs that activate their target promoter, resulting in significant luciferase activity on a medium copy plasmid (> 10,000-fold over background). Thus, in order to avoid oversaturation of the cell with ECFs, we relied on this basal activity of the P_*BAD*_ promoter of ECF expression, assaying the luciferase signal from the ECF target promoter for 8 h and inducing AS expression after 2 h from the beginning of the measurement (Fig. 6). In plasmid-encoded circuits, we observed a decrease of the luciferase signal (80-fold for AS16, Fig. 6b; 160-fold for AS22, Fig. 6g) in both AS-switches, suggesting that in both cases AS factors bind the cognate ECF σ factor and thereby lower the target promoter activity. However, upon the production of the AS factor, in both circuits we also noted a marked decrease in the OD_600_ values (5-fold when compared to the uninduced strain; Fig. 6d,i). Hence, the induction of these AS factors, when encoded on medium copy number plasmids, lead to toxic effects on the cells. Therefore, it is not possible to discriminate if the reduction of luciferase signal is due to the inhibiting activity of the AS on the ECF σ factor, or to the AS overexpression that causes a growth defect in the strain analyzed. However, when examining the same ECF σ/AS circuits integrated into the chromosome, we also observed a significant decrease in the luciferase signal (30-fold for AS16, Fig. 6c; 150-fold for AS22, Fig. 6h), while showing almost identical OD_600_ values of AS-induced and uninduced cultures (Fig. 6e,l). These results suggest that in both chromosomally integrated circuits, the AS factors retained their ability to sequester the cognate ECF σ factor, while the toxicity effects observed on medium copy number plasmids were completely abolished. Therefore, we conclude that for these particular AS factor-based circuits, the chromosomal integration represents the optimal configuration to modulate the behavior of ECF-σ switches, while keeping the cells viable. Our experiments also showed that it is possible to use CRIMoClo plasmid to efficiently generate and integrate multiple large constructs (∼10 kb) in parallel, starting from MoClo library parts. Finally, since CRIMoClo plasmids are fully compatible with the MoClo standard, it is possible to easily generate, test and compare the same genetic circuit in a plasmid, or chromosomal configuration.

## Conclusions

One of the aims of synthetic biology is the design of complex genetic pathways. In such pathways, optimizing the balance in production of different pathway components is often a key step to reach the desired output product. To do so, one of the strategies consists in adjusting the transcription and the translation rate of each gene in the pathway. Using a combinatorial framework like the MoClo system allows for the generation of a library of interchangeable parts (such as promoters, ribosome binding sites, and transcriptional regulators), that can be combined in several permutations in order to find the right balance of gene expression levels that feature functional synthetic circuit activity. With the aim of increasing the spectrum of possible permutations in synthetic circuit generation, the CRIMoClo system designed here represents an additional degree of freedom to the possible configurations of a given synthetic circuit. In particular, the system supports the generation of genetic circuits from reusable, MoClo-compatible parts and their integration into 4 orthogonal *att* sites in the genome of *E. coli.* By combining the properties of the CRIM plasmids with those of the MoClo system, the framework allows for easy generation and rapid integration of large synthetic constructs. In the present work we have assembled and integrated constructs up to ~ 10 kb with high efficiency. Given that under natural conditions the recombination machinery is capable of integrating typical phage genomes of ~ 50 kb, the manipulation and integration of larger synthetic circuits should, in principle, be possible. However, at such sizes a finite plasmid stability and other factors might limit transformation and thus integration efficiency, and further studies are required to probe the upper limits of the approach.

Utilizing four different resistance modules, CRIMoClo system facilitates fast and reliable multiple integrations. Moreover, the modular design of CRIMoClo plasmids allows for an easy, further expansion of the system for compatibility with other Modular Cloning-based frameworks, such as CIDAR MoClo [15], Start-Stop Assembly [41], Loop Assembly [42] and Mobius Assembly [43]. With these features the CRIMoClo system brings the combinatorial assembly to the next step, enabling a seamless transition between plasmid-encoded and chromosomally integrated genetic circuits. Finally, with the CRIMoClo system, it is possible to generate and simultaneously utilize a combination of plasmid-borne and chromosomally integrated genetic modules. Therefore, when used together, the different gene copy numbers implemented in the MoClo and CRIMoClo systems enable adjustment of gene expression levels over wide ranges, thereby facilitating, e.g., the optimization of enzyme expression levels in a biosynthetic pathway in order to maximize downstream product formation.

## Materials and methods

### Bacterial strains and growth conditions

*E. coli* strains used in this study are listed in Additional file 2: Table S1. The strains were cultivated in LB (LB Broth Miller, Sigma Aldrich Cat.No. L3522) medium or MOPS minimal medium (TEKNOVA Cat.No. M2106; 0.5% glycerol as carbon source) at 37 °C shaking at 250 rpm. To maintain plasmids, the following antibiotics were used: chloramphenicol at 25 μg/ml, kanamycin at 50 μg/ml, spectinomycin at 100 μg/ml, gentamicin at 10 μg/ml. For the selection of single-copy integrants, antibiotics were added as follows: chloramphenicol at 6 μg/ml, kanamycin at 10 μg/mL, spectinomycin at 35 μg/ml, gentamicin at 5 μg/ml. For the blue-white screening, LB plates containing isopropyl β-D-1-thiogalactopyranoside (IPTG) at 0.1 mM and 5-Bromo-4-chloro-3-indolyl-β-D-galactoside (X-Gal) at 40 μg/ml were used.

### Molecular biology techniques

Oligonucleotides were provided by Sigma-Aldrich. PCR reactions were performed using Q5 High-Fidelity DNA Polymerase (New England Biolabs) or Taq DNA Polymerase (New England Biolabs). Reaction mixtures were purified using the E.Z.N.A. Cycle-Pure Kit (Omega Bio-Tek). For gel extraction, Zymoclean Gel DNA Recovery Kit (Zymo Research) was used. Type IIs restriction enzymes (*BpiI* and *BsaI*) and T4 DNA ligase were purchased from Thermo Scientific. DNA sequence verification was performed by Eurofins Genomics. Transformation of different chemically competent *E. coli* strains was performed according to the Inoue method [44] or using the transformation and storage solution (TSS) technique [25] (see below).

### CRIMoClo vector construction

CRIMoClo vectors generated in this study (Additional file 3: Table S2.1) were assembled using Ligase Cycling Reaction (LCR) [10] and Gibson Assembly [9]. For the construction of the first 8 CRIMoClo vectors (Level M and P with chloramphenicol resistance and 4 *att* sites), DNA fragments were PCR-amplified using Q5 High-Fidelity DNA Polymerase (New England Biolabs) with the primers listed in Additional file 4: Table S3. In particular, the *tL3* terminator together with one of four different phages attachment sites (*att*_HK022_, *att*_P21_, *att*_ϕ80_, *att*_λ_) was amplified using the universal forward primer GF0524 in combination with the reverse primers GFC0525, GFC0526 and GFC0530, using CRIM plasmids pAH68, pAH81 and pAH153 [23] as templates, respectively. The amplification of attλ from pAH120 [23] was performed in was performed in two steps, using primers GFC0524/GFC0527 and GFC0528/GFC0529 to remove an undesired *BpiI* restriction site. The γ conditional origin of replication of R6K was amplified from pAH68 [23] using the primers GFC0531/GFC0532, while the chloramphenicol resistance cassette was amplified from pKD3 [17] using the primers GFC0533/GFC0534. The rgnB terminator was amplified from pAH68 [23] using the primers GFC0535/GFC0536. Finally, the MoClo multicloning region was amplified with the primers GFC0537/GFC0538 using pSVM-mc [4] and pICH82094 [13] as Level M and Level P templates, respectively. The generated fragments (blunt-end and 5′ phosphorylated) were fused via Ligase Cycling Reaction (LCR) according to de Kok et al. [10]. In particular, a reaction mix of 0.3 U Taq DNA Ligase (New England Biolabs), 3 nM DNA parts, 30 nM bridging oligos (Additional file 4: Table S3), and 8% (v/v) DMSO was used under the following assembly protocol: 2 min at 94 °C, then 50 cycles of 10 s at 94 °C, 30 s at 60 °C, and 60 s at 65 °C, followed by incubation at 4 °C. The newly generated set of plasmids (Additional file 3: Table S2.1) were used as templates for the assembly of the next 8 CRIMoClo plasmids featuring the kanamycin resistance cassette, using Gibson Assembly [9]. In particular, the backbones from pSV004, pSV006, pSV008, pSV077, pSV016, pSV018, pSV080 and pSV079 (Additional file 3: Table S2.1) and the kanamycin cassette from pSVM-mc [4] were PCR-amplified using Q5 High-Fidelity DNA Polymerase (New England Biolabs) using the primers GF0807/GF0532 and GF0805/GF0808. The generated fragments were fused via Gibson Assembly [9], setting a reaction in which 50 ng of backbone DNA (0.03 pmol) were mixed with 0.09 pmol of insert (represented by the resistance cassette) and Gibson Reaction Mix (New England Biolabs) in a final volume of 20 μl. The Gibson Assembly was performed for 1 h at 50 °C. The resulting plasmids were then used as templates to generate eight gentamicin and eight spectinomycin resistant CRIMoClo plasmids, using Gibson Assembly [9]. In particular, the backbones pSV125, pSV126, pSV127, pSV128, pSV219, pSV220, pSV221 and pSV222 were PCR amplified using Q5 High-Fidelity DNA and the primers GF0945/GF0532 in order to maintain the promoter of the kanamycin cassette. These fragments were then fused with the spectinomycin and gentamicin coding sequences (amplified from pMA333 [20] and pABC2 [45] using the primers GF0858/GF0947 and GF0856/GF0949, respectively) via Gibson Assembly, following the protocol described above. The plasmid maps of all 32 CRIMoClo vectors can be found in Additional file 5.

### Modular cloning (MoClo) reactions

The integrative CRIMoClo-based plasmids generated in this study (Additional file 3: Table S2.2) were assembled on CRIMoClo vectors, using MoClo-compatible parts listed in Additional file 3: Table S2.2. MoClo reactions were set up using 15 fmol of each DNA part (PCR product or plasmid), 1 μl of the required restriction enzyme (*BsaI* or *BpiI*), 1 μl of T4 DNA Ligase (5 U/μl) and 2 μL of T4 DNA Ligase buffer (10x), in a final reaction volume of 20 μL. The reaction was incubated in a thermocycler for 5 h at 37 °C, 10 min at 50 °C and 10 min at 80 °C. Then 2 μl of the reaction mixture was added to 50 μl chemically competent DH5α λpir cells, incubated for 30 min on ice and transformed by heat shock. This was followed by adding 950 μl of liquid LB to the transformation, and cells were recovered for 45 min at 37 °C. Finally, 40 μl of the transformation was plated on selective LB plates and emerging colonies were verified by colony PCR and restriction digestion.

### CRIMoClo plasmid integration using competent cells pre-transformed with the helper plasmid

The integration of the CRIMoClo plasmids was performed similarly as described by Haldimann and Wanner [23]. In particular, 2 μl of purified plasmid was added to 50 μl chemically competent *E. coli* SV01 cells, carrying one of the CRIM helper plasmids (pAH69, pAH121, pINT-ts and pAH123 [23]). The cells were incubated for 30 min on ice and transformed by heat shock. Then 950 μl of liquid LB was added to the transformation mix, and cells were incubated at 37 °C for 1 h and at 42 °C for 30 min (to induce the phage-derived integrase (*int*) gene and simultaneously cure the helper plasmid). Then 80 μl of the transformation mixture were spread onto selective agar plates and incubated at 37 °C overnight. Colonies were tested by colony PCR using the primers P1–P2–P3–P4 (Table 1 and Additional file 4: Table S3), pre-cultured once in non-selective medium and then tested for antibiotic resistance for stable integration and loss of the helper plasmid.

### CRIMoClo plasmid integration using TSS competent cells

As an alternative way to achieve chromosomal integration (e.g. in the multi-integration experiment) we prepared competent cells using the TSS method [25]. A single clone of *E. coli* SV01 was picked from LB agar plates and pre-cultured in 3 ml LB media at 37 °C, shaking at 250 rpm. When the OD_600_ reached 0.5–0.8, cells were chilled in ice for 10 min and then 500 μl of cell culture were mixed with 500 μl of TSS 2x and left in ice for 45 min. Subsequently, 50 μg of purified CRIMoClo-based plasmid and 50 μg of the cognate helper plasmid were added to the cell mixture and left in ice for 45 min, followed by 1 h at 30 °C and 30 min at 42 °C shaking at 250 rpm. Then 200 μl of the cell culture was plated on selective plates and grown overnight at 37 °C. In the case of strains possessing multiple resistance cassettes, we selected only for resistance encoded by the latest integrated construct. The obtained colonies were tested by colony PCR using the primers P1–P2–P3–P4 (Table 1 and Additional file 4: Table S3), pre-cultured once in non-selective medium and then tested for antibiotic resistance for stable integration and loss of the helper plasmid.

### Integration stability assay

To assay the stability of the P_*BAD*_-lux construct integrated into the four selected *att* sites, the strains GFC0214, GFC0216, GFC0218, GFC0500 (Additional file 2: Table S1) were streaked onto an LB agar plate without antibiotic selection. The same strains were also streaked onto LB agar plates supplied with 0.2% arabinose to induce luciferase expression. The plates were grown overnight at 37 °C and subsequently screened for light production imaging them using a BioRad Chemidoc MP imaging system, in presence and absence of bright light (Additional file 1: Figure S6A). To assay the stability of the P_*BAD*_-lux construct integrated into bacterial genome, a single colony of each strain was grown in liquid LB medium without antibiotic selection for 7 days, re-inoculating the cultures in fresh medium every 24 h. Aliquots of the liquid cultures (5 μl) were then streaked onto LB agar plates and LB agar plates supplied with 0.2% arabinose (both without antibiotic selection) and grown overnight at 37 °C. Subsequently, the plates were screened for luciferase activity as described above (Additional file 1: Figure S6B).

### Microplate reader assays

Microplate reader assays were performed as follows. For each *E. coli* strain, a single bacterial colony was picked from selective plates and grown in liquid LB medium until stationary phase (37 °C shaking at 250 rpm; 7–8 h). These pre-cultures were diluted 1:6000 into MOPS minimal medium and grown overnight (37 °C shaking at 250 rpm) until they reached an OD_600_ of 0.5–0.6. The cultures were then diluted to an OD_600_ of 0.05 in fresh MOPS minimal medium (see above) and 100 μl of culture were loaded in the wells of a black 96-well plate (GREINER catalog no.: 655097). Using a Tecan Infinite F200 pro microplate reader the plate was incubated for 10 h (37 °C with shaking) and OD_600_ as well as luminescence and fluorescence were measured every 5 min (10 min in case of multiple fluorescence/luminescence measurements). For switching the circuits from the OFF to the ON state, after 2 h of incubation cells were induced with the appropriate inducer (arabinose, ATc, IPTG) at the concentrations indicated in the Figures and incubation was resumed.

### Luciferase bleed-through correction

The luciferase activity for each replicate in Fig. 3, Fig. 4, Fig. 5 and Fig. 6 was determined as follows. First, the raw luminescence data obtained from microplate reader measurements were background-corrected by subtracting luminescence values obtained from a control well containing the growth medium alone. Then we corrected for luminescence bleedthrough (i.e. light-scattering) from neighboring wells on the microplate, by using a de-convolution algorithm developed in our group [46]. Last, the resulting values were divided by the optical density at each time point during the course of the experiment, which yields the luciferase activity in relative luminescence units per OD_600_ (RLU/OD).

## Supplementary information

**Additional file 1: Figures S1 - S11.**

**Additional file 2: Table S1.** Bacterial strains used in this study.

**Additional file 3: Table S2.** CRIMoClo vectors generated in this study.

**Additional file 4: Table S3.** Primers used in this study.

**Additional file 5. **Plasmid maps of the 32 CRIMoClo plasmids.

## Data Availability

All data generated or analyzed during this study are included in this published article (and its supplementary information files). Bacterial strains and plasmids are available from the authors upon reasonable request.
