# Supplementary material for: CRIMoClo plasmids for modular assembly and orthogonal chromosomal integration of synthetic circuits in Escherichia coli
Source: J Biol Eng. 2019 Nov 28;13:92. doi: 10.1186/s13036-019-0218-8 (PMC6883643; doi:10.1186/s13036-019-0218-8)
Supplement: Supplementary file 1 — Additional file 1: Figures S1 - S11. [file 13036_2019_218_MOESM1_ESM.pdf]

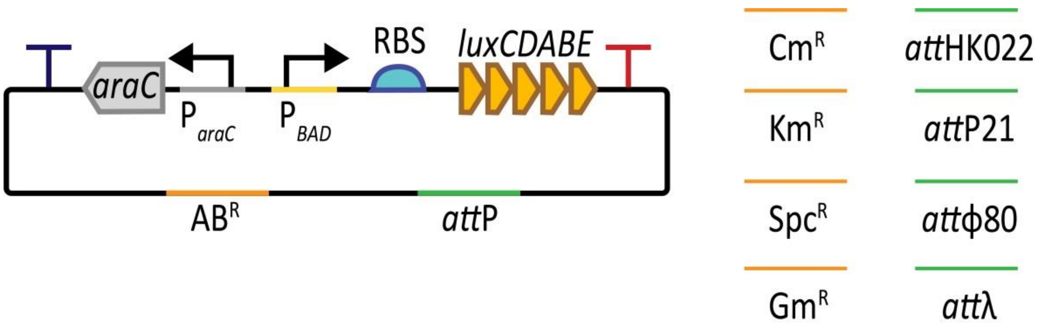

Figure S1. Representation of the P_{BAD}-*lux* construct assembled on 16 CRIMoClo plasmids. Four level 0 parts consisting in a P_{BAD} promoter [26] in combination with the AraC repressor encoded in divergent orientation, a strong ribosome binding sequence (st8 [28]), a luciferase cassette from *Photorhabdus luminescens* [27] and a strong synthetic terminator (L3S2P21) [29], were fused together into a level 1 recipient vector and subsequently subcloned into 16 CRIMoClo vectors.

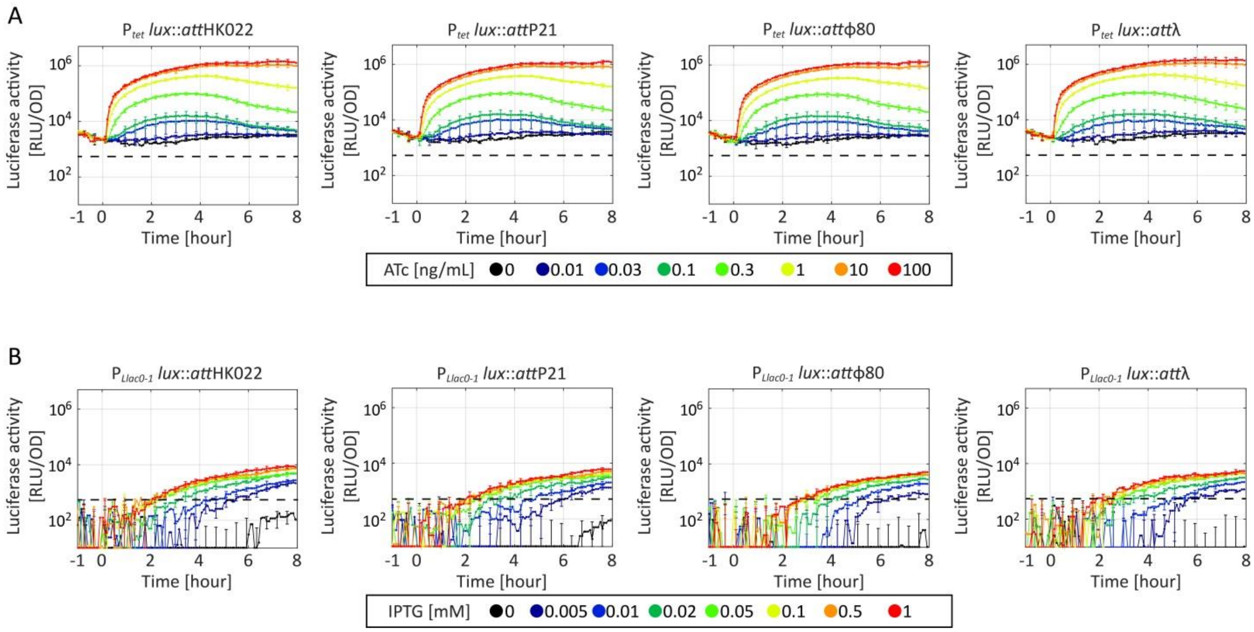

Figure S2. Comparison of dynamical response of luciferase activity of the P_{tet}-*lux* (A) and $P_{l0\text{-}1}$-*lux* (B) constructs integrated into att_{HK022}, att_{P21}, $att_{\phi 80}$, att_{λ}, after the addition of indicated concentrations of inducer at t=0 h. The results are averaged from at least two independent biological assays and error bars denote standard deviations. The black dashed line represents the instrument detection limit, defined as three times the standard deviation of the luminescence signal registered in a well filled with media, divided by the averaged OD_{600} values registered in the same well.

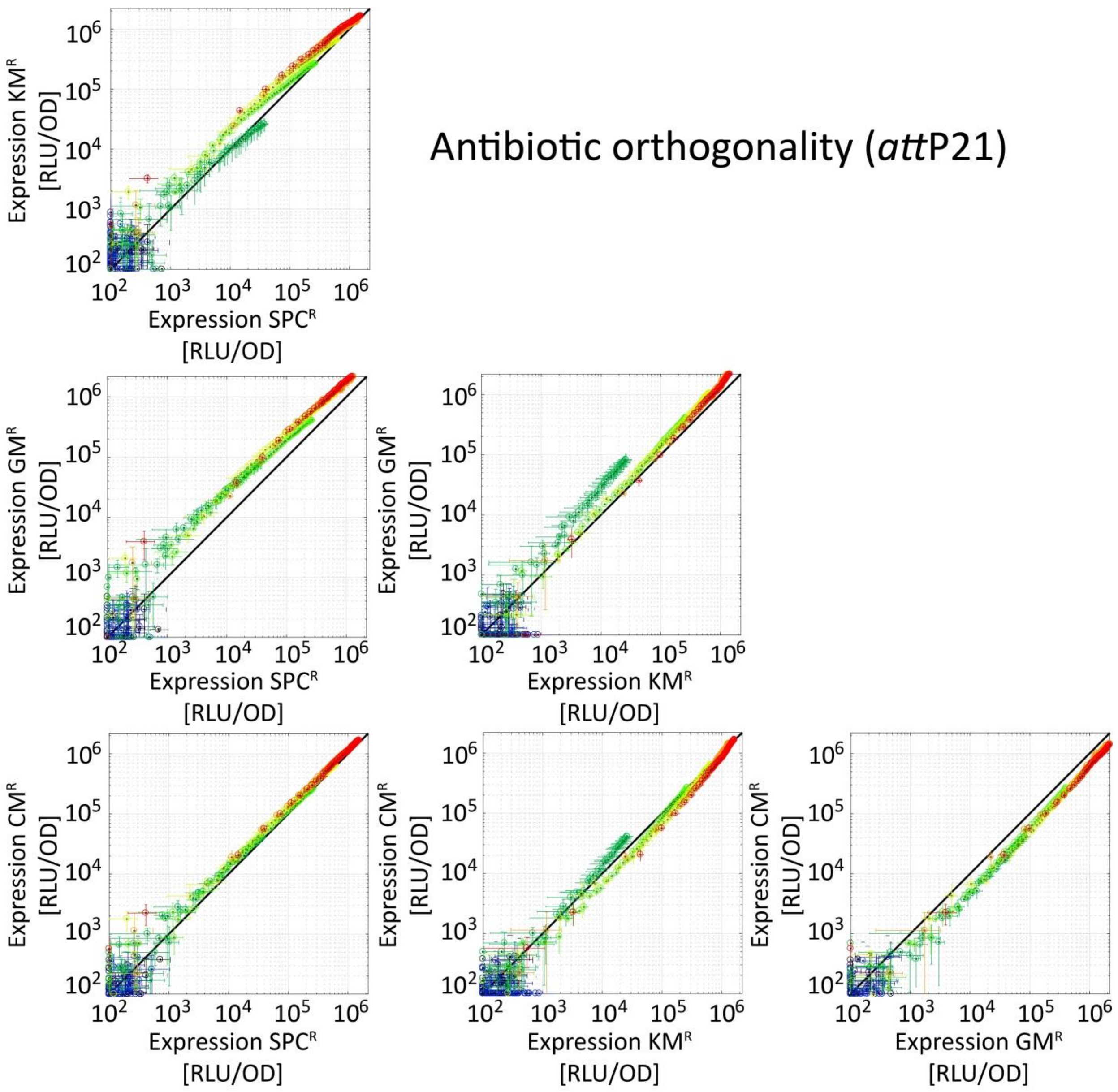

Figure S3. Orthogonality of reporter gene expression between resistance cassettes used for integration in the phage P21 *att* site. Correlation graphs between luciferase activities obtained from P_{BAD}-*lux* integrated into att_{P21}, using CRIMoClo plasmids with four indicated resistance cassettes (chloramphenicol, kanamycin, spectinomycin, gentamicin). All data indicate averages from at least two independent biological assays and error bars denote standard deviations.

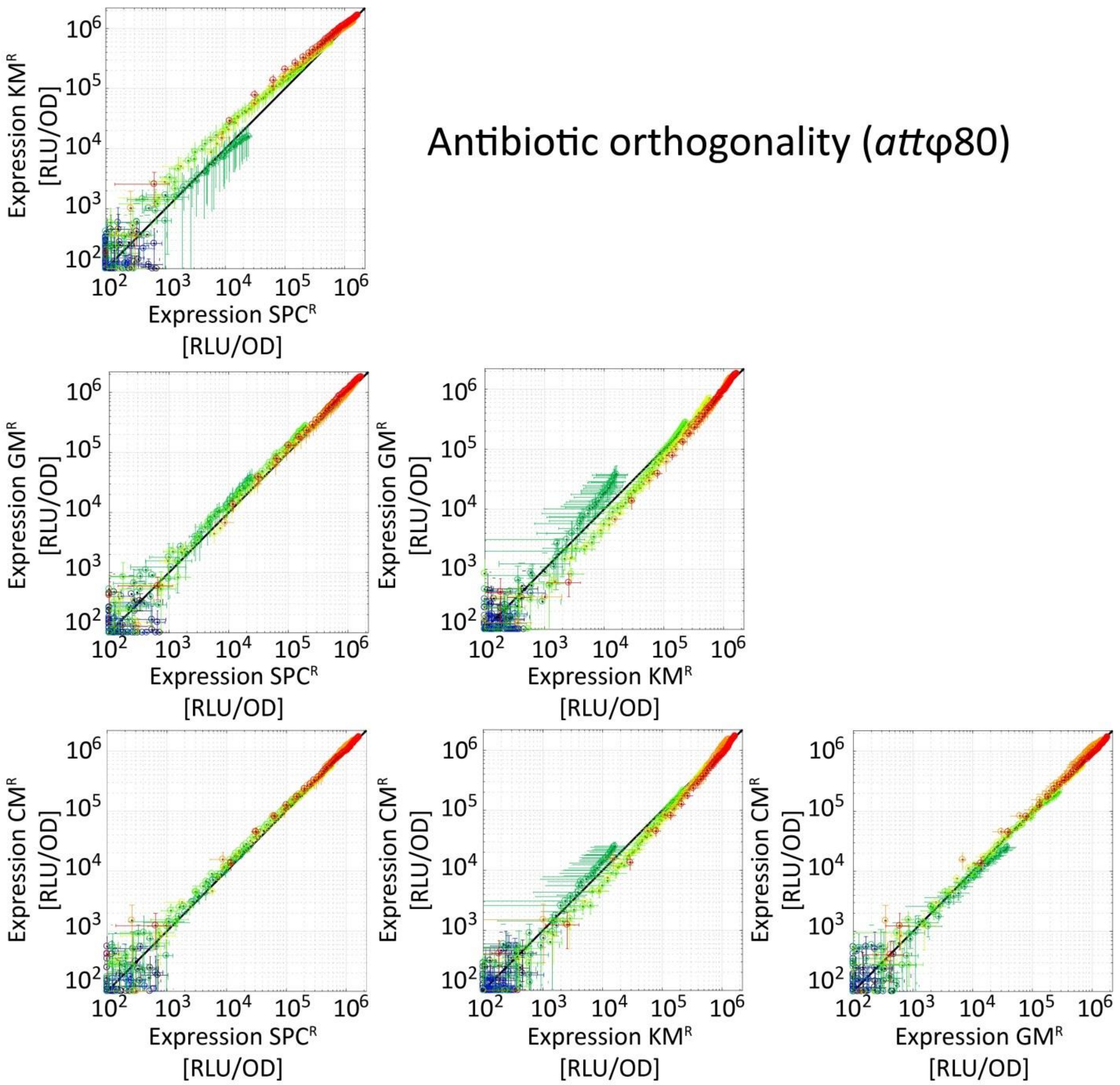

Figure S4. Orthogonality of reporter gene expression between resistance cassettes used for integration in the phage ϕ80 *att* site. Correlation graphs between luciferase activities obtained from P_{BAD}-*lux* integrated into *att*$_{\phi 80}$, using CRIMoClo plasmids with four indicated resistance cassettes (chloramphenicol, kanamycin, spectinomycin, gentamicin). All data indicate averages from at least two independent biological assays and error bars denote standard deviations.

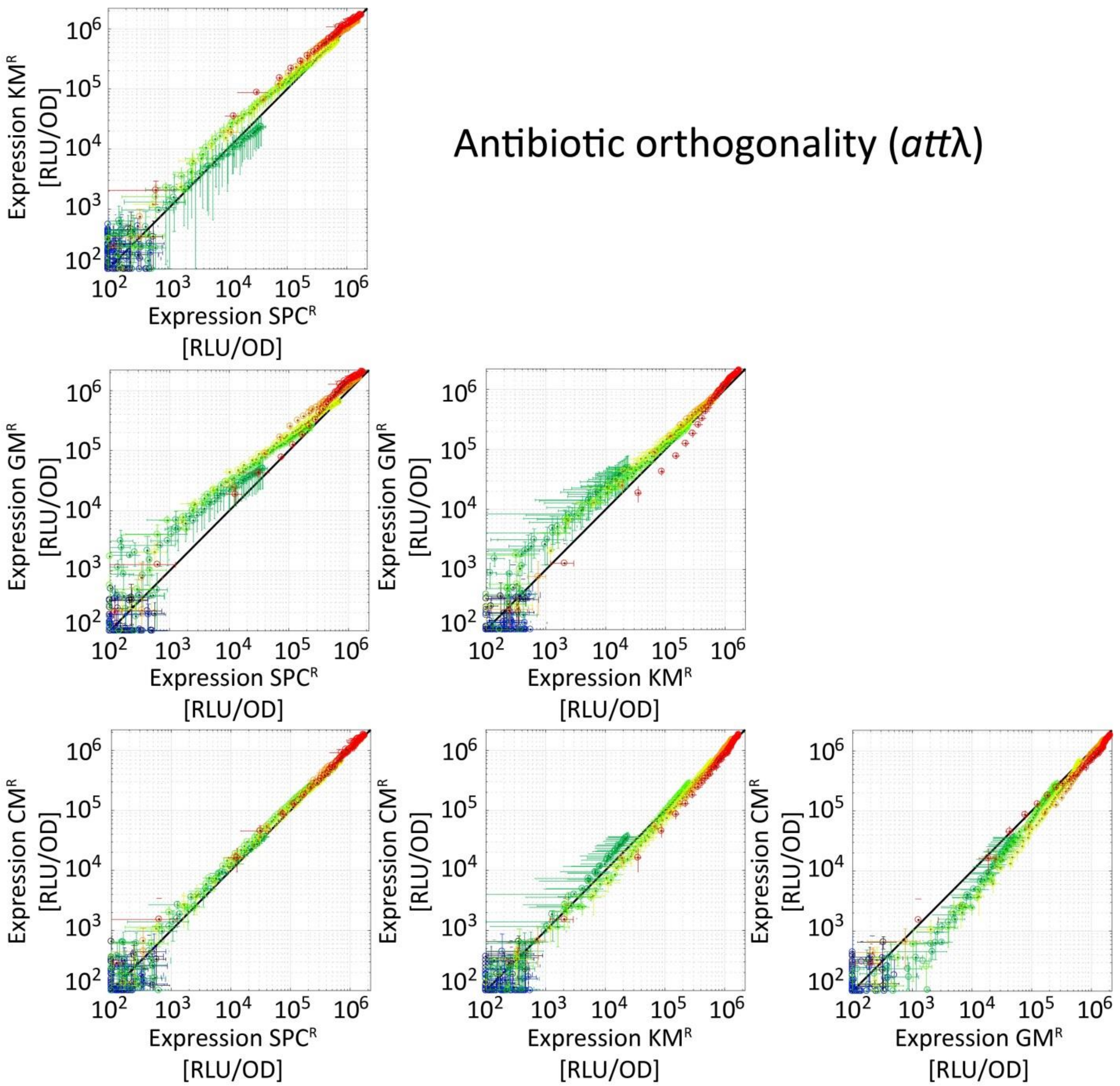

Figure S5. Orthogonality of reporter gene expression between resistance cassettes used for integration in the phage λ *att* site. Correlation graphs between luciferase activities obtained from P_{BAD}-*lux* integrated into att_{λ}, using CRIMoClo plasmids with four indicated resistance cassettes (chloramphenicol, kanamycin, spectinomycin, gentamicin). All data indicate averages from at least two independent biological assays and error bars denote standard deviations.

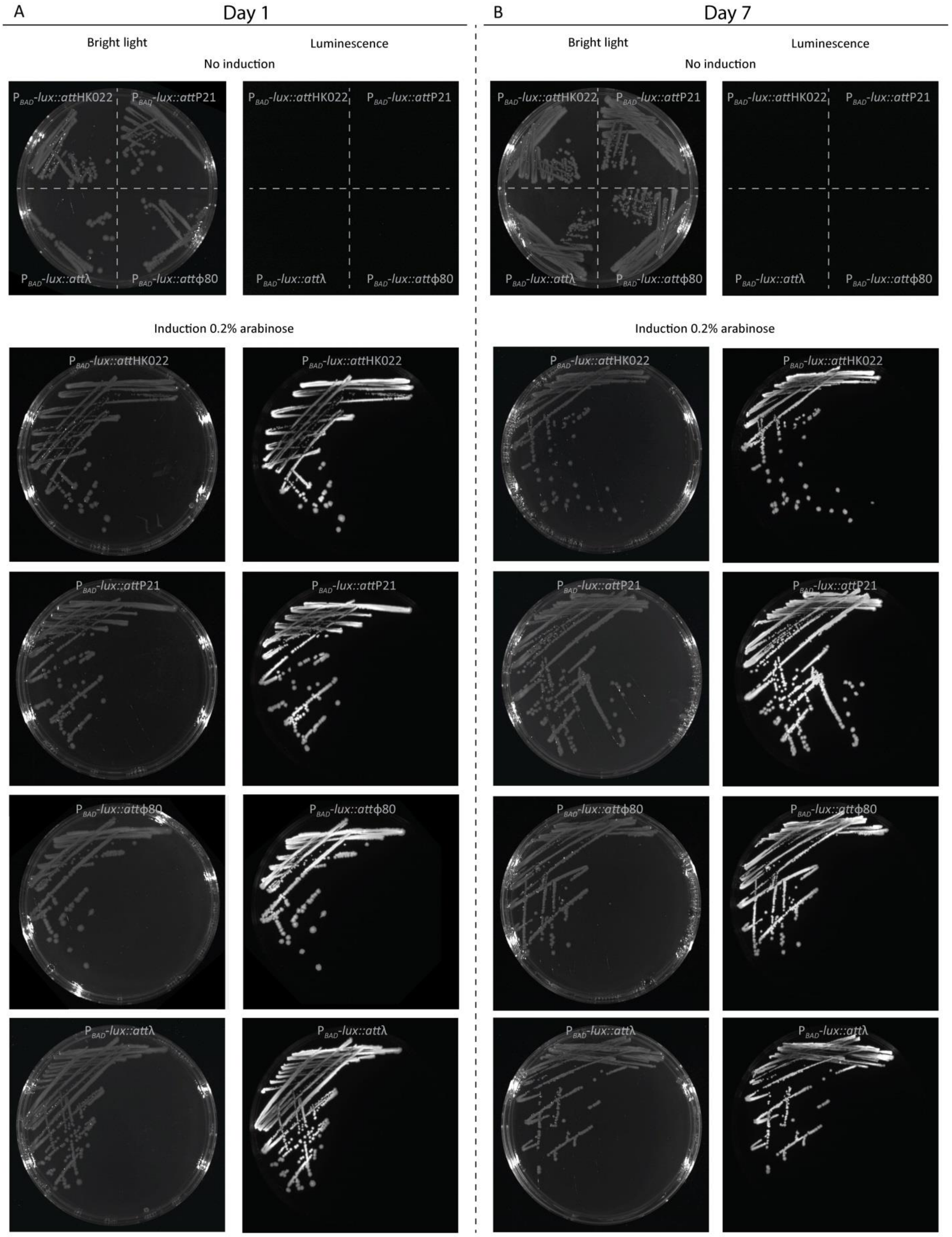
A
Day 1
B
Day 7
Bright light
Luminescence
No induction
P_{BAD}-lux::attHK022
P_{BAD}-lux::attP21
P_{BAD}-lux::attλ
P_{BAD}-lux::attϕ80
Induction 0.2% arabinose
P_{BAD}-lux::attHK022
P_{BAD}-lux::attP21
P_{BAD}-lux::attϕ80
P_{BAD}-lux::attλ

Figure S6. Integration stability assay of P_{BAD}-*lux* construct integrated into the four phage attachment sites. The strains GFC0214, GFC0216, GFC0218, GFC0500 (Supplementary Table S1) were streaked onto LB agar plates in absence and presence of inducer and imaged to screen light activity. (A) Bacterial strains streaked from glycerol stocks onto LB agar plates without antibiotic selection. (B) Same bacterial strains displayed in (A) streaked onto LB agar plates without antibiotic selection after beeing precultured in LB medium for 7 days (refreshing the cultures every 24 h) without antibiotic selection.

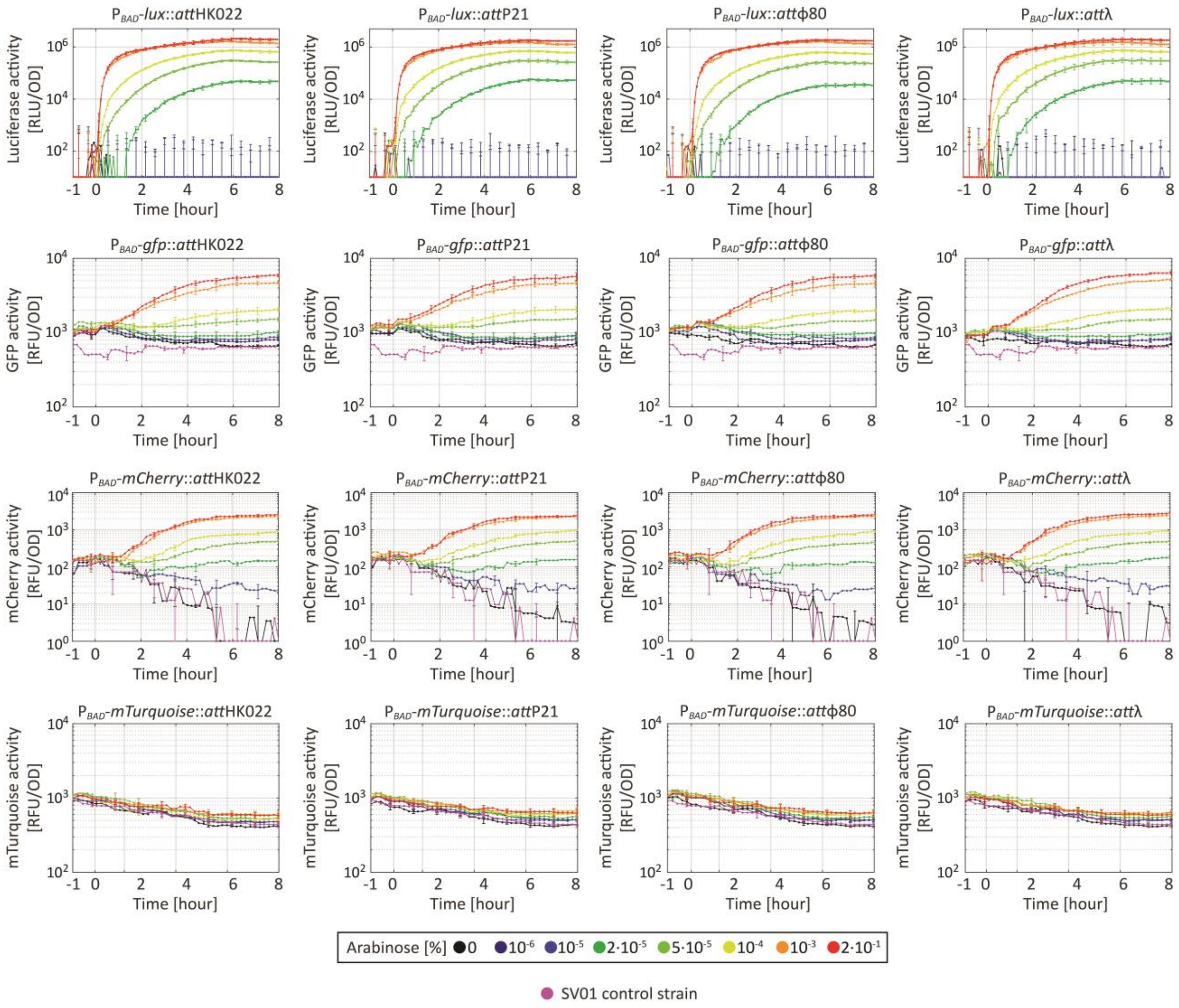

Figure S7. Comparison of dynamical response of the indicated reporters activity fused with the P_{BAD} promoter and integrated into the indicated *E. coli att* sites, after the addition of indicated concentrations of arabinose at t=0 h. The results are averaged from at least two independent biological assays and error bars denote standard deviations.

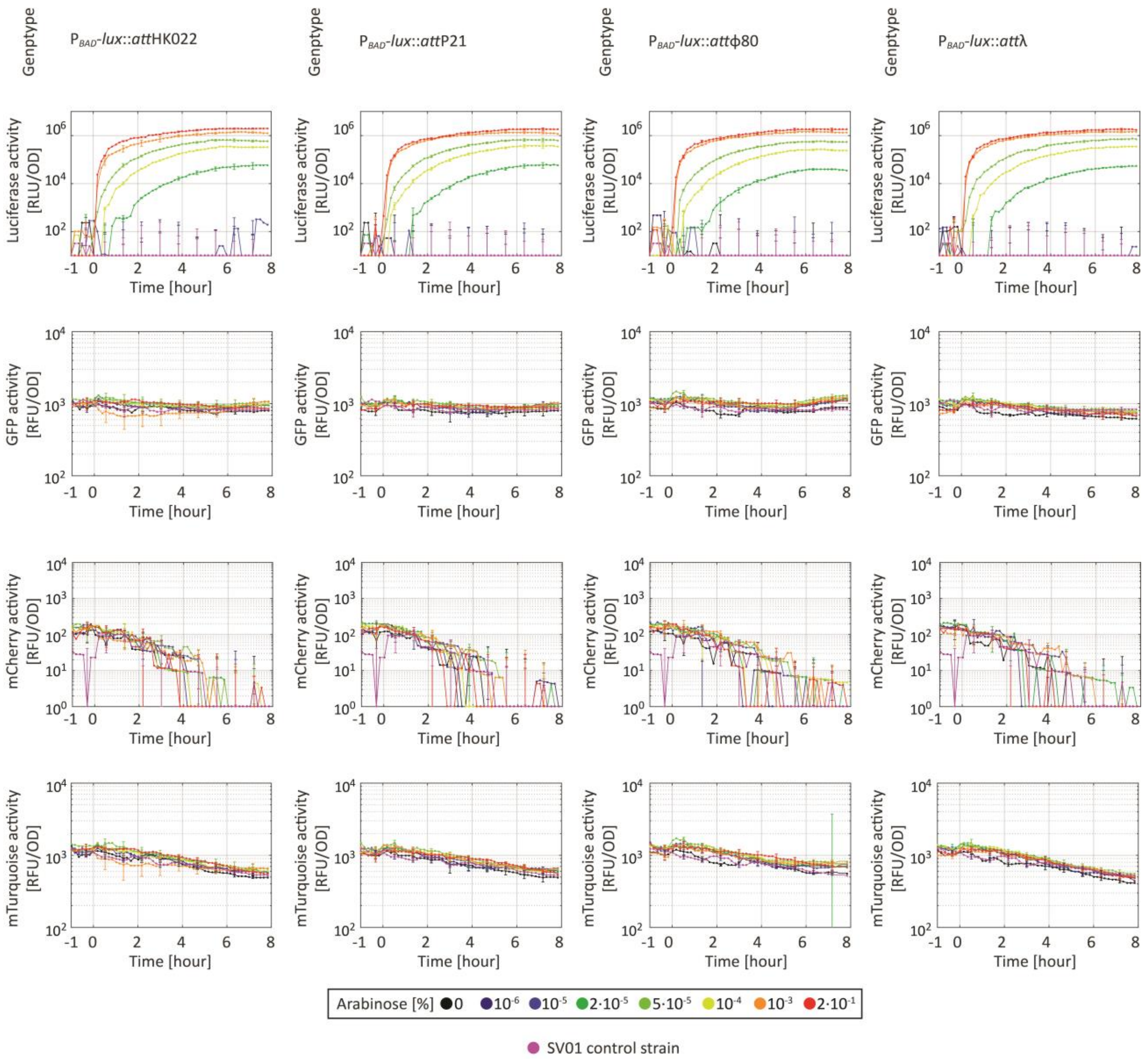

Figure S8. Comparison of dynamical response of the indicated reporters activity in *E. coli* strains harbouring a P$_{BAD}$-*lux* construct integrated into the indicated *att* sites, after the addition of indicated concentrations of arabinose at t=0 h. The results are averaged from at least two independent biological assays and error bars denote standard deviations.

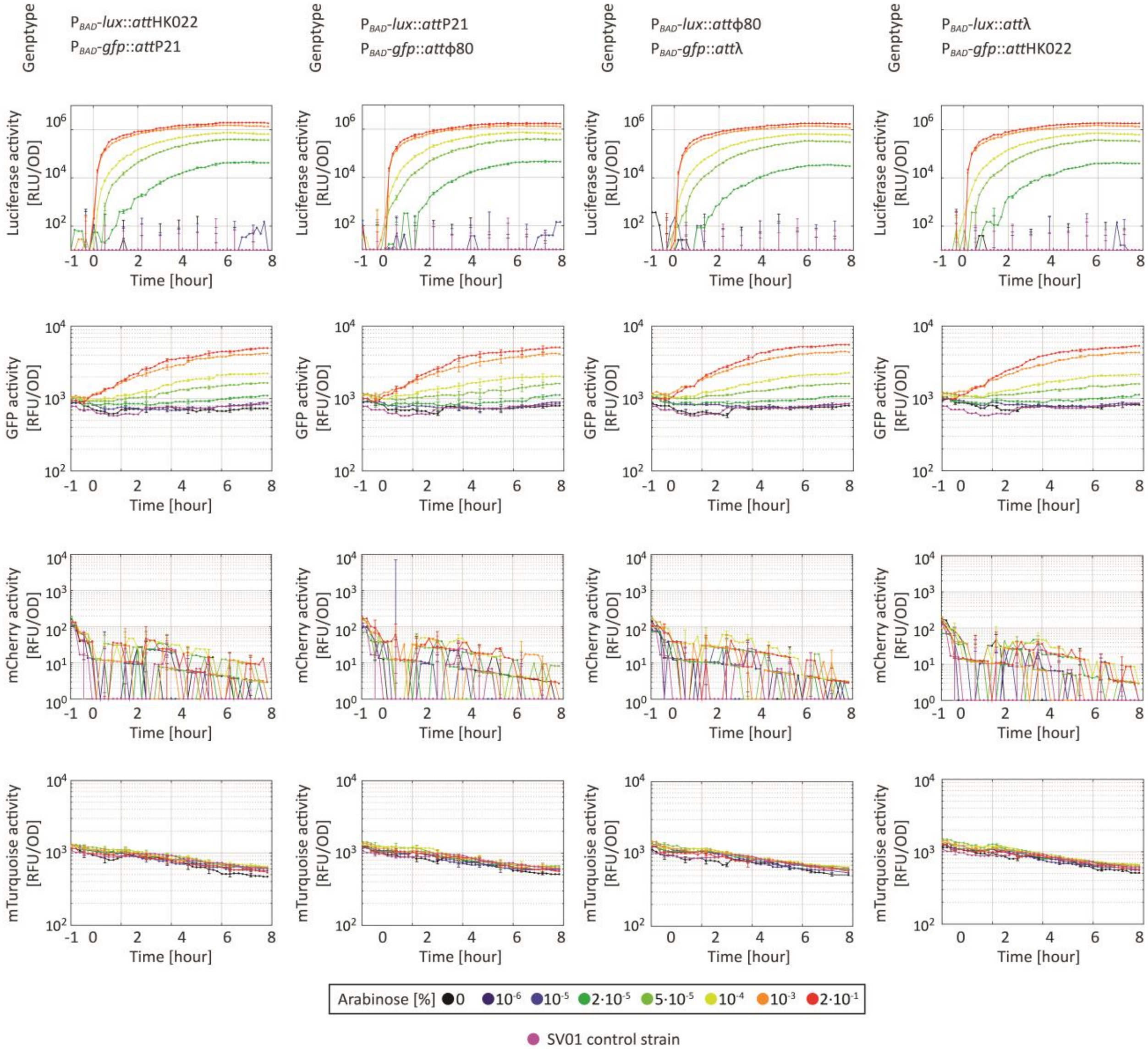

Figure S9. Comparison of dynamical response of the indicated reporters activity in *E. coli* strains harbouring a P_{BAD}-*lux* and a P_{BAD}-*gfp* construct integrated into the indicated *att* sites, after the addition of indicated concentrations of arabinose at t=0 h. The results are averaged from at least two independent biological assays and error bars denote standard deviations.

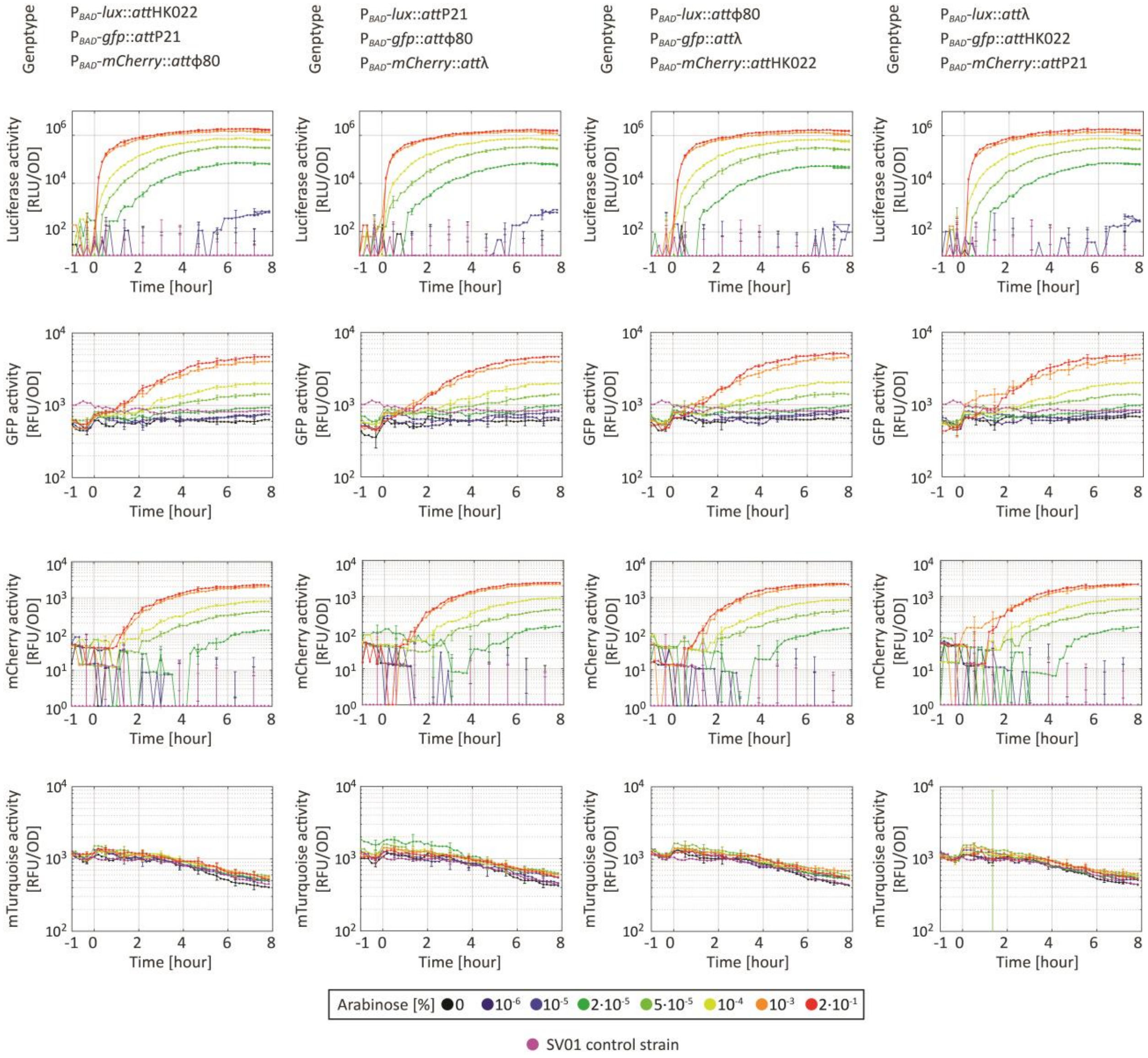

Figure S10. Comparison of dynamical response of the indicated reporters activity in *E. coli* strains harbouring a P_{BAD}-*lux*, a P_{BAD}-*gfp* and P_{BAD}-*mCherry* construct integrated into the indicated *att* sites, after the addition of indicated concentrations of arabinose at t=0 h. The results are averaged from at least two independent biological assays and error bars denote standard deviations.

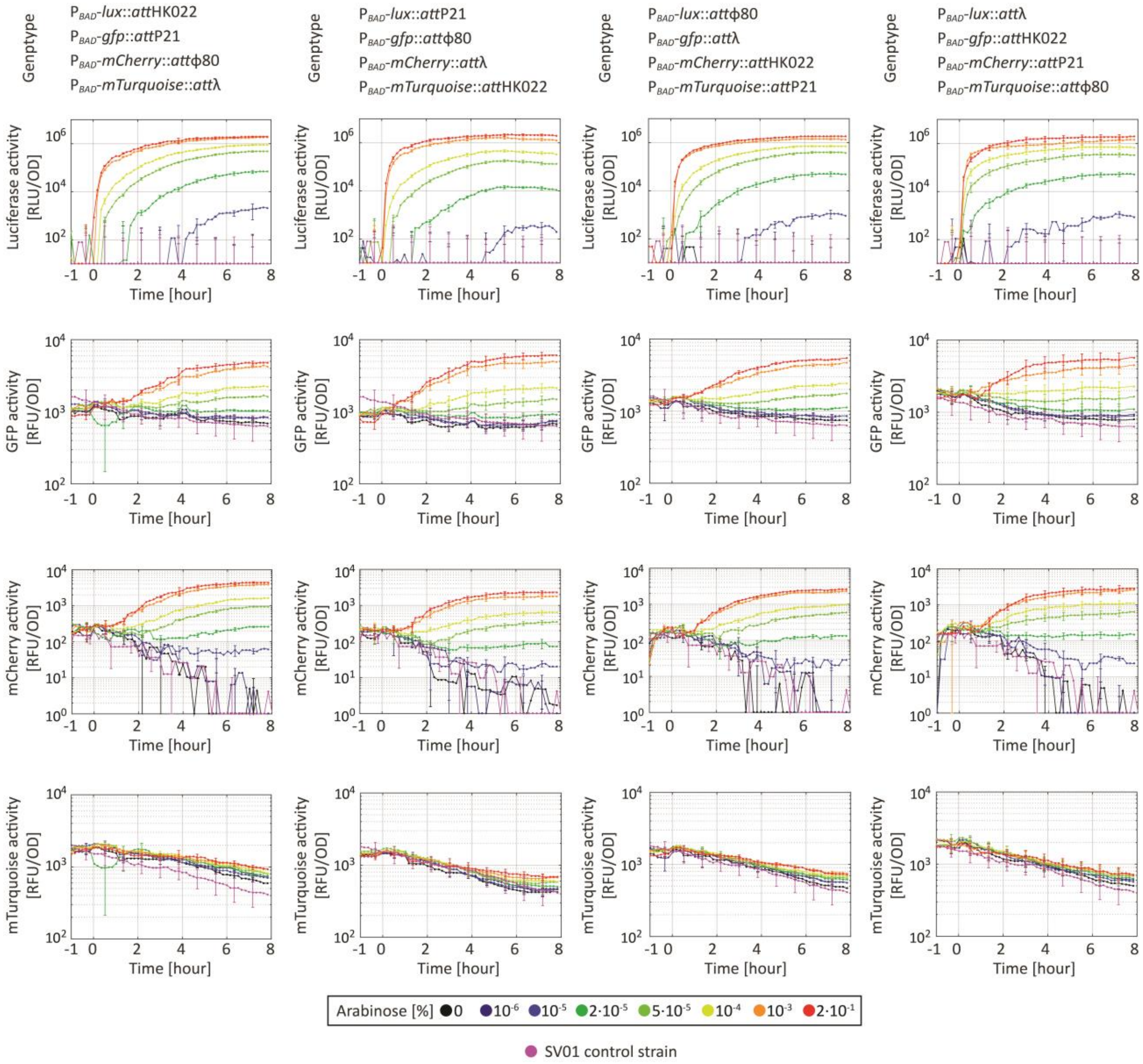

Figure S11. Comparison of dynamical response of the indicated reporters activity in *E. coli* strains harbouring a P_{BAD}-*lux*, a P_{BAD}-*gfp*, a P_{BAD}-*mCherry* and a P_{BAD}-*mTurquoise* construct integrated into the indicated *att* sites, after the addition of indicated concentrations of arabinose at t=0 h. The results are averaged from at least two independent biological assays and error bars denote standard deviations.
